# Rhizoctonia Bataticola Lectin (RBL) Induces Caspase-8-Mediated Apoptosis in Human T-Cell Leukemia Cell Lines but Not in Normal CD3 and CD34 Positive Cells

**DOI:** 10.1371/journal.pone.0079311

**Published:** 2013-11-14

**Authors:** Radha Pujari, Sachin M. Eligar, Natesh Kumar, Srikanth Barkeer, Vishwanath Reddy, Bale M. Swamy, Shashikala R. Inamdar, Padma Shastry

**Affiliations:** 1 National Centre for Cell Science, Pune University Campus, Pune, Maharashtra, India; 2 Department of Biochemistry, Karnatak University, Dharwad, Karnataka, India; Columbia University, United States of America

## Abstract

We have previously demonstrated immunostimulatory activity of a fungal lectin, Rhizoctonia bataticola lectin (RBL), towards normal human peripheral blood mononuclear cells. The present study aimed to explore the anticancer activities of RBL using human leukemic T-cell lines, Molt-4, Jurkat and HuT-78. RBL exhibited significant binding (>90%) to the cell membrane that was effectively inhibited by complex glycoproteins such as mucin (97% inhibition) and asialofetuin (94% inhibition) but not simple sugars such as N-acetyl-D-galactosamine, glucose and sucrose. RBL induced a dose and time dependent inhibition of proliferation and induced cytotoxicity in the cell lines. The percentage of apoptotic cells, as determined by hypodiploidy, was 33% and 42% in Molt-4 and Jurkat cells, respectively, compared to 3.11% and 2.92% in controls. This effect was associated with a concomitant decrease in the G0/G1 population. Though initiator caspase-8 and -9 were activated upon exposure to RBL, inhibition of caspase-8 but not caspase-9 rescued cells from RBL-induced apoptosis. Mechanistic studies revealed that RBL induced cleavage of Bid, loss of mitochondrial membrane potential and activation of caspase-3. The expression of the anti-apoptotic proteins Bcl-2 and Bcl-X was down regulated without altering the expression of pro-apoptotic proteins- Bad and Bax. In contrast to leukemic cells, RBL did not induce apoptosis in normal PBMC, isolated CD3+ve cells and undifferentiated CD34+ve hematopoietic stem and progenitor cells (HSPCs). The findings highlight the differential effects of RBL on transformed and normal hematopoietic cells and suggest that RBL may be explored for therapeutic applications in leukemia.

## Introduction

Cell surface glycans are involved in the regulation of tumor progression, proliferation, invasion and metastasis [1], [2]. Due to aberrant glycosylation, tumor cells display carbohydrate profiles on the cell surface that are different from those of non-transformed cells. Lectins have unique affinities to carbohydrates and hence the binding properties of lectins have been used to detect sugar moieties on normal and transformed cell surfaces and study the structural and functional role of cell surface carbohydrates [3], [4]. Lectins are reported to induce cytotoxicity or inhibition of growth in various cancer cells [5], [6]. The two main properties of lectins- selectivity and cytotoxicity- have, therefore, been exploited for devising therapeutic strategies against cancer.

Extensive research has been carried out to investigate the cytotoxic properties of plant and animal lectins [7], [8]. Two cytotoxic isolectins -KML-IIU and KML-IIL isolated and characterized from Korean mistletoe exhibit cytotoxicity in various human and mouse cancer cell lines [7]. Wheat germ lectin (WGA) is another cytotoxic lectin with deleterious effect on the viability of H3B (human hepatocellular carcinoma), JAr (human choriocarcinoma) and ROS (rat osteosarcoma) cell lines [8]. Galectins are the most widely studied animal lectins and are demonstrated to affect survival, signal transduction, and proliferation in many cancers particularly in colorectal cancers [9], [10]. Achatinin, a lectin from hemolymph of snail, *Achatina fulica* is highly cytotoxic against MCF7, a human mammary carcinoma cell line [11]. Musca Domestica Larva Lectin (MLL) has been shown to inhibit cell proliferation and induce apoptosis of human hepatoma BEL-7402 [12]. More recently, fungal lectins have gained importance largely due to the discovery that some of these lectins exhibit potent antitumor activities. A number of lectins from mushrooms such as Inocybe umbrinella lectin isolated from the fruiting body of a toxic mushroom, *Inocybe umbrinella*, is reported to inhibit proliferation of hepatoma HepG2 cells and breast cancer MCF7 cells [13]. Agrocybe aegerita lectin exhibits anticancer activity against HeLa cells [14] and a lectin from *Pleurotus citrinopileatus* exhibits anti-tumor activity in mice bearing sarcoma S180 and hepatoma H-22 cells [15]. Though the anti-tumor properties of many fungal lectins have been reported, the precise mechanism of action has not been studied.

We have earlier reported that RBL, a lectin isolated from phytopathogenic fungus *Rhizoctonia bataticola* has exclusive specificity for complex high mannose type N-linked glycans including tri- and tetra- antennary high mannose oligosaccharide [16]. RBL exhibited mitogenic activity in human PBMC and stimulated the production of Th1/Th2 cytokines via activation of p38 MAPK and STAT-5 signaling pathways [17]. We had also demonstrated that RBL exerts its effect in normal PBMC by binding to CD45, a receptor-like protein tyrosine phosphatase [18]. The present study was undertaken to investigate the anticancer properties of RBL against leukemic T-cells.

## Materials and Methods

### Ethics Statement

The study was approved by the ethics committee of NCCS. Written informed consent was obtained from the volunteers. The CD34+ve hematopoietic stem and progenitor cells (HSPCs) isolated from human umbilical cord blood was a kind gift from Dr. Lalitha Limaye, NCCS, these samples were procured for a project that was approved by the institutional ethics committee.

### Isolation and Purification of RBL

Isolation, purification and characterization of RBL from fungal mycelia has been described previously [16].

### Cell Culture

Human leukemic cell lines Molt-4, Jurkat and HuT-78 were procured from American Type Culture Collection (ATCC Rockville, USA) and maintained in RPMI 1640 (Gibco, USA) supplemented with 10% heat inactivated fetal calf serum (FCS), 100 µg/ml streptomycin and 100units/ml penicillin at 37°C in 5% CO2 and 95% humidified air. The cell lines were split every alternate day.

Human PBMC were isolated by density centrifugation of heparinized blood of healthy donors using Histopaque 1077 (Sigma Chemicals, USA). T-cells were isolated by sorting of PBMC using phycoerythrin-labeled CD3 antibody (BD Bioscience, USA) by flow cytometry. The purity of T-cells obtained post-sorting was >95%. The cells were cultured in RPMI 1640 medium supplemented with 10% heat inactivated fetal calf serum (FCS), 100 µg/ml streptomycin and 100units/ml penicillin. CD34+ve hematopoietic stem and progenitor cells (HSPCs) were isolated from human umbilical cord blood and expanded *in vitro* according to protocol reported earlier [19]. The cells were maintained in IMDM medium supplemented with 20% FCS and 25000 cells were seeded per well in 96 well tissue culture plates.

### Analysis of Lectin Binding

Molt-4 and Jurkat cells were incubated with FITC-conjugated RBL (1 µg/100 µl) for 1 h at 4°C. After washing, the percentage of cells positive for RBL binding was determined using flow cytometry analysis. To determine the localization of RBL, cells were stained with FITC-RBL at 4°C, fixed with 2% para-formaldehyde, mounted on slides and visualized by confocal laser scanning microscope (Zeiss LSM 510, Germany) equipped with 488nm and 560nm Argon lasers.

Sugar specificity of RBL was determined by pre-incubating FITC-RBL with 10 µg/100 µl of mucin, asialofetuin, N-acetyl –D-galactosamine (GalNAc) glucose and sucrose (Sigma chemicals, USA) for 1 h at room temperature. This lectin-sugar complex was added to the PBMC preparation and analyzed by flow cytometry.

### Tritiated Thymidine Incorporation Assay

Human leukemia cell lines- Molt-4, Jurkat and normal T-cells isolated from PBMC were seeded 25000 cells/100 µl in 96 well tissue culture plate (BD Falcon). The cells were stimulated with RBL (2.5 and 5 µg/ml) for 72 h. During the last 18 h, tritiated thymidine 1 µCi/well (Board of Radiation and Isotope Technology, India) was added and the stimulation was measured as counts per minute (CPM).

### MTT Assay

Cells seeded in a 96 well plate were treated with RBL in serial concentrations (0.625 to 5 µg/ml) for different time points (12 to 48 h) in a humidified atmosphere (37°C, 5% CO2). At the end of the treatment, 0.5 mg/ml of MTT (Sigma chemicals, USA) was added and cells were lysed with 10% SDS in 0.01 N HCl and absorbance was measured at 570 nm with reference wavelength of 640nm. The absorbance value of untreated cells at each time point was considered as 100 to calculate the percent viable cell number. For determining the involvement of caspases in RBL-induced apoptosis the cells were pretreated with the following inhibitors- Z-VAD-FMK- a pan-caspase inhibitor, Z-IETD-FMK- caspase-8 inhibitor and Z-LEHD-FMK- caspase-9 inhibitor (BD Biosciences, USA) followed by MTT assay.

### Cell Cycle Analysis

Cells were treated with RBL (5 µg/ml) for 24 h, washed and fixed in 70% chilled ethanol for 30 min at 4°C followed by incubation with 50 µl Ribonuclease A (5 mg/ml in PBS, DNase free) for 10 min at room temperature and stained with Propidium Iodide (50 µg/ml in PBS). The DNA content was analyzed on the FL-2A channel of flow cytometer (FACS Calibur, BD Biosciences, USA) equipped with a 488 nm argon laser on a linear scale for cell cycle analysis. The data was analyzed by Cell Quest Pro software for determining the distribution of cells in different phases of cell cycle.

### Detection of Apoptosis by Annexin-V Staining

Cells, treated with RBL (5 µg/ml) for 6, 12 and 24 h were harvested and resuspended in binding buffer. The cell suspension was incubated with FITC Annexin-V (BD Biosciences, USA) and propidium iodide for 15 min at room temperature in dark followed by flow cytometry analysis. The percentage of cells positive for Annexin-V, PI alone and in combination was calculated by dot plot analysis using Cell Quest Pro software.

### Activation of Caspases

Activation of caspases −8 and −9 were determined using colorimetric assay kit (Novagen, USA) according to manufacturer’s instructions. The fold increase in caspase activity was calculated with respect to untreated control.

For detection of caspase-3 activation, cells were treated with RBL (5 µg/ml) for 24 h and active caspase was determined by caspase-3 detection kit (Calbiochem, USA) according to manufacturer’s instructions. Briefly, cells were washed with washing buffer and incubated with 5 µl of FITC-DEVD-FMK for 30 min. Flow cytometry analysis was done to measure FITC- positive cells in control and treated samples.

### Determination of Mitochondrial Membrane Potential (MMP)

Alterations in mitochondrial membrane potential in RBL treated Molt-4 cells was assessed by the uptake of Tetramethylrhodamine ethyl ester (TMRE) (Invitrogen, USA). Molt-4 cells were washed in PBS and incubated in freshly diluted (1∶1000) dye at 37°C in 5% CO2 for 30 min. The cells were washed, resuspended in the incubation buffer, and analyzed immediately on the FL-2 channel of a flow cytometer. Decrease in the red fluorescence intensity was considered as an indication of the breakdown in the membrane potential.

### Western Blotting

Cells were treated with RBL (5 µg/ml) for different time intervals up to 12 h. At specific time intervals, the cells were lysed using RIPA lysis buffer (120 mM NaCl, 1.0% Triton X-100, 20 mM Tris–HCl, pH 7.5, 100% glycerol, 2 mM EDTA, protease inhibitor cocktail, Roche, Germany). Total protein was electrophoresed on SDS-polyacrylamide gels and blotted onto PVDF membrane (Millipore, USA). After blocking with 5% BSA, the blots were probed with antibodies to Bid, Bcl-2, Bcl-X, Bad and Bax (BD Biosciences, USA). The bands were visualized by chemiluminescence using SuperSignal West Femto Maximum Sensitivity Substrate (Pierce, USA). Actin (MP biomedicals, USA) was used as loading control. PARP cleavage, was determined in lysates of cells treated with different concentrations of RBL (1.25–5 µg/ml) by western blot analysis using anti-PARP antibody (Santacruz, USA).

### Statistical Analysis

Statistical analysis was performed using Student's t test and Mann–Whitney rank sum test. A p-value <0.05 was considered to be statistically significant.

## Results

### Carbohydrate Dependent Binding of RBL to Leukemic Cell Lines

**Figure 1:**
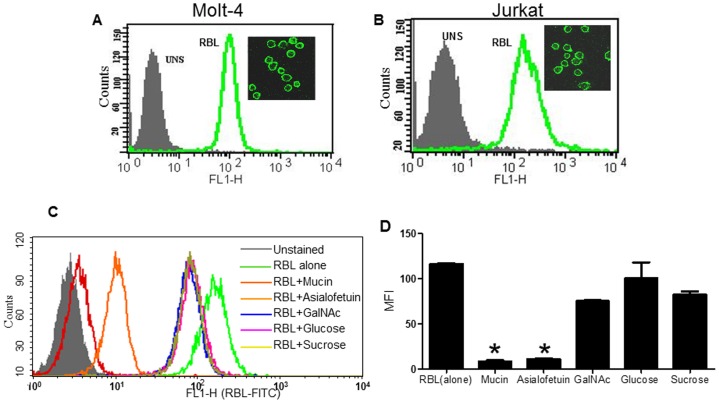
Binding of RBL and inhibition with sugars. The binding of FITC- labeled RBL to Molt-4 (A) and Jurkat (B) cells was determined by flow cytometry analysis. The histoplots depict the profiles of unstained cells (shadow) and cells stained with FITC-RBL (green). The binding of RBL to cell membrane of Molt-4 (A inset) and Jurkat (B inset) was visualized by confocal laser scanning microscopy. Original magnification 60×. (C) Molt-4 cells were stained with FITC-labeled RBL alone or RBL pre-incubated with different sugars and flow cytometry analysis was performed. X-axis represents fluorescence intensity, Y-axis represents cell number. The overlay shows profiles of the unstained cells (shadow), RBL stained cells (green) and cells stained with RBL +Mucin (red), +asialofetuin (orange), +GalNAc (blue), +glucose (pink), and sucrose +(yellow). (D) The graph represents mean MFI±SE from three independent experiments. *p<0.05 difference in MFI compared to cells stained with RBL alone.

RBL exhibited intense binding (>90% positivity) to Molt-4 and Jurkat cells (Fig. 1A and B). Localization studies were done by confocal microscopy by incubating the cells with FITC RBL at 4°C. As expected, RBL was specifically observed on the cell membrane (Fig. 1A and B Inset) indicating high expression of RBL binding receptors on the cell surface.

The carbohydrate specificity of RBL was determined by pre-incubating FITC-RBL with 10 µg/100 µl of mucin, asialofetuin, N-acetyl –D-galactosamine (GalNAc) glucose and sucrose. The lectin-sugar complex was added to the Molt-4 cells and binding was analyzed by flow cytometry (Fig. 1C). As depicted in the graph (Fig. 1D), 99.99% of cells were positive for RBL binding with an MFI of 120. Mucin exhibited the maximum inhibition (97%) of RBL binding followed by asialofetuin (94% inhibition), but simple sugars tested at the same concentration did not significantly inhibit RBL binding suggesting that RBL binds to complex sugars on the surface of leukemic cells.

### Anti-proliferative and Cytotoxic Activity of RBL

**Figure 2:**
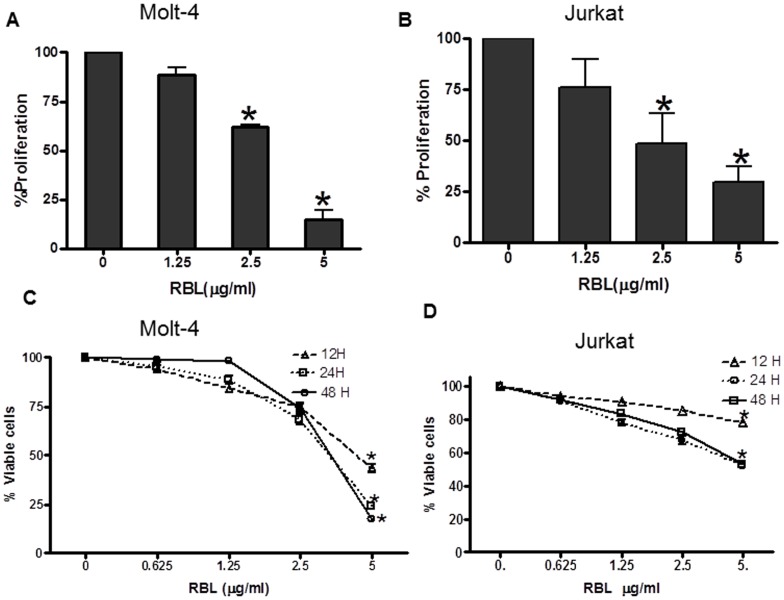
Effect of RBL on proliferation and viability of leukemic cell lines-. Molt-4 (A) and Jurkat (B) cells were exposed to serial concentrations of RBL for 72 h and proliferation was determined by tritiated thymidine incorporation assay. The percentage cell proliferation was calculated by considering the counts per minute of untreated control cells as 100. Molt-4(C) and Jurkat (D) cell lines were exposed to serial concentrations of RBL for different time periods and cell viability was assessed by MTT assay. The absorbance value of untreated cells was considered as 100 to calculate percent viable cell number. The values presented in the graph are mean±SE of three independent experiments done in triplicates. *p<0.05 difference compared to untreated cells.

The dose response effect of RBL on proliferation of leukemic cell lines was determined by a tritiated thymidine incorporation assay. RBL induced 40% and 75% inhibition of proliferation at 2.5 and 5 µg/ml concentration, respectively, in Molt-4 cells (Fig. 2A). At the same concentration, RBL induced 30% and 60% inhibition of proliferation, respectively, in Jurkat cells (Fig. 2B). A putative cytotoxic effect of RBL on Molt-4 and Jurkat cell lines was examined by the MTT assay. Time course and dose response experiments using Molt-4 cells revealed that exposure to RBL at a dose of 5 µg/ml for 12 h resulted in 40% viability, which further decreased to 25% and 13% at 24 and 48 h respectively (Fig. 2C). Jurkat cells showed 70% cell viability at 12 h and 50% viability at 24 h and 48 h of RBL treatment (Fig. 2D). These preliminary data suggested that RBL exhibits cytotoxic activity killing human leukemia cells.

### Induction of Apoptosis by RBL

**Figure 3:**
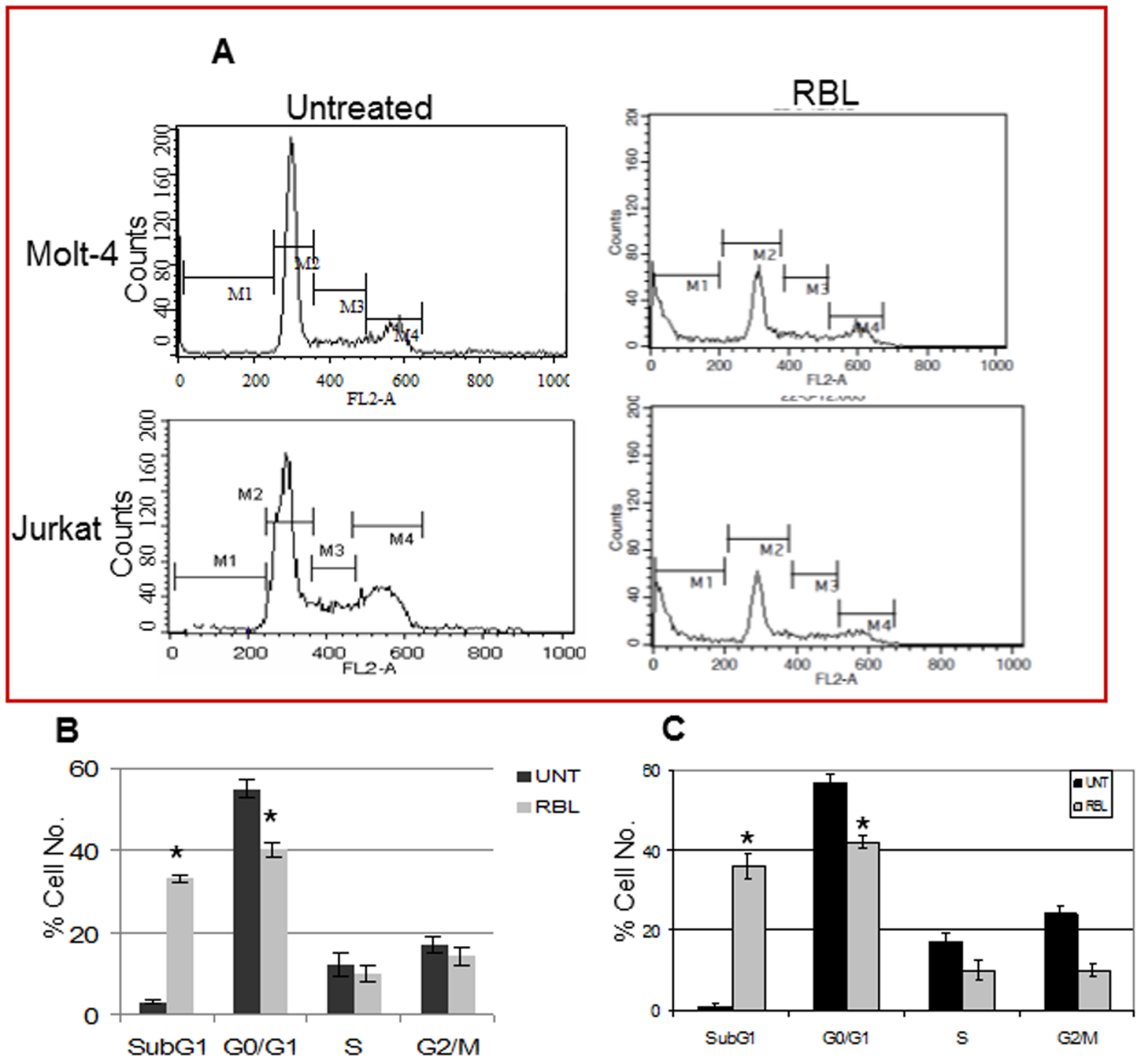
Effect of RBL on different phases of cell cycle. (A) Molt -4 and Jurkat cells, treated with RBL, were stained with PI and acquired on FL2-A channel of flow cytometer equipped with 488nm laser. The X-axis represents the DNA content of the cells and the Y-axis represents the cell number. The graph depicts the percentage of Molt-4 (B) and Jurkat (C) cells in different phases of cell cycle. The data is mean ±SE of three independent experiments. *p<0.05 significant difference between untreated and RBL treated cells.

The distribution of cells in the different phases of cell cycle was determined by exposing Molt-4 and Jurkat cells to RBL(5 µg/ml) for 24 h followed by propidium iodide (PI) staining and flow cytometric analysis (Fig. 3A). The graphs revealed a significant increase in the hypo-diploid population with 33% and 42% as compared to 3.11% and 2.92% in controls among Molt-4 (Fig. 3B) and Jurkat cells (Fig. 3C), respectively. This was associated with a decrease in the G0/G1population with 30.05 and 20.75% in comparison to 54.88 and 56.76% in controls among Molt-4 (Fig. 3B) and Jurkat cells (Fig. 3C) respectively. The reduction in the G1 population may be due to apoptotic cell loss.

**Figure 4:**
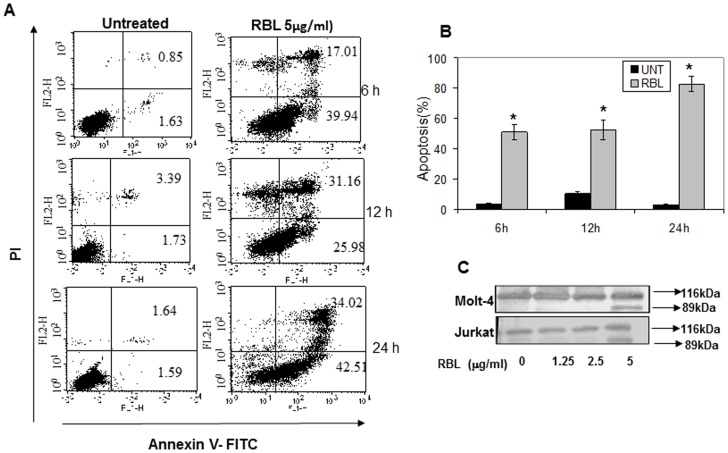
RBL induces apoptosis of Molt-4 and Jurkat cells. (A) Molt-4 cells were treated with RBL (5 µg/ml) for 6, 12 and 24 h followed by Annexin- V-FITC and PI staining. The X-axis depicts Annexin-V positive cells and Y- axis depicts PI positive cells. The numbers in each quadrant represent percent positive cells. Dot plots are representative of three similar experiments. (B) The graph represents % apoptosis (Annexin-V-positive cells) mean±SE of three independent experiments. *p<0.05 indicates significant difference between treated and untreated cells. (C) Whole cell lysates of Molt-4 and Jurkat cells were exposed to RBL (1.25 to 5 µg/ml) for 24 h and probed with anti-PARP antibody. The blot shows total and cleaved PARP. The data is representative of two similar experiments.

The apoptotic potential of RBL was quantified by Annexin-V PI staining, to distinguish viable, early and late apoptotic or necrotic cells. As shown in Fig. 4A, RBL treatment resulted in a significant increase in Annexin-V positive-PI negative cells within 6 h of treatment (39.94%). There was also a time dependent increase in Annexin-V and PI positive cells from 17.01% at 6 h to 31.16% at 12 h and a marginal increase to 34.02% at 24 h, indicating that RBL induces apoptosis of Molt-4 cells. However, there is a small population of PI- positive Annexin -V negative cells at 12 h of RBL exposure suggesting that RBL induced necrosis at later time points. The graph (Fig. 4B) depicts the percentage of apoptotic cells (Annexin-V positive) after RBL exposure for different time periods.

The effect of RBL on PARP cleavage was determined by western blot analysis. A 89kDa cleaved fragment was observed in Molt-4 and Jurkat cells treated with RBL (5 µg/ml) for 24 h. No cleavage was observed at doses of 1.25 and 2.5 µg/ml suggesting that 5 µg/ml is minimal effective concentration for inducing cell death (Fig. 4C). These results suggested that the cytotoxic activity of RBL may be attributed to induction of apoptosis in Molt-4 and Jurkat cells.

### RBL Activates Extrinsic and Intrinsic Apoptotic Pathways

**Figure 5:**
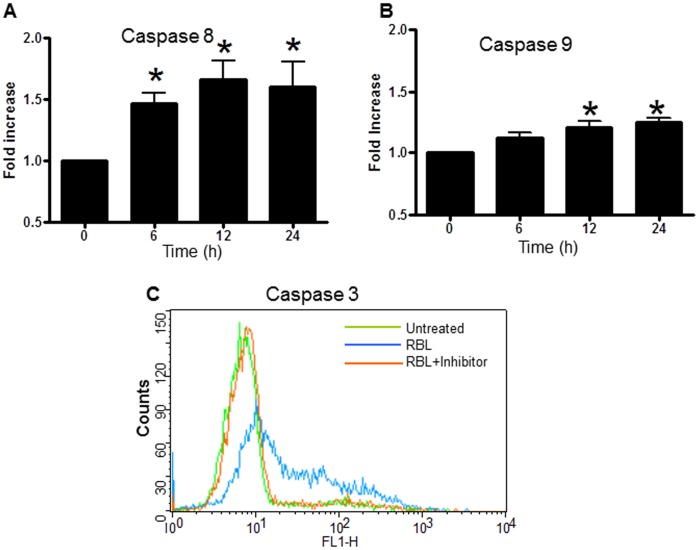
Effect of RBL on activation of caspases. Molt-4 cells were treated with 5 µg/ml RBL for different time periods and activities of caspase-8 (A) and caspase-9 (B) were assessed by colorimetric assay using specific substrates Ac-IETD-pNa and Ac-LEHD-pNa respectively. The graph depicts fold increase in caspase activity with respect to 0 h controls (activity at 0 h was considered as 1). The data is mean±SE values from three independent experiments. *p value <0.05 in comparison with 0 h controls. (C) Molt-4 cells were treated with RBL (5 µg/ml) for 12 h and activity of caspase-3 was determined by flow cytometry analysis using FITC tagged caspase–3 inhibitor (blue line). Cells pretreated with caspase-3 inhibitor ZVAD-FMK (red line) and untreated cells (green line) were used as controls. The histoplot is representative of three similar experiments.

**Figure 6:**
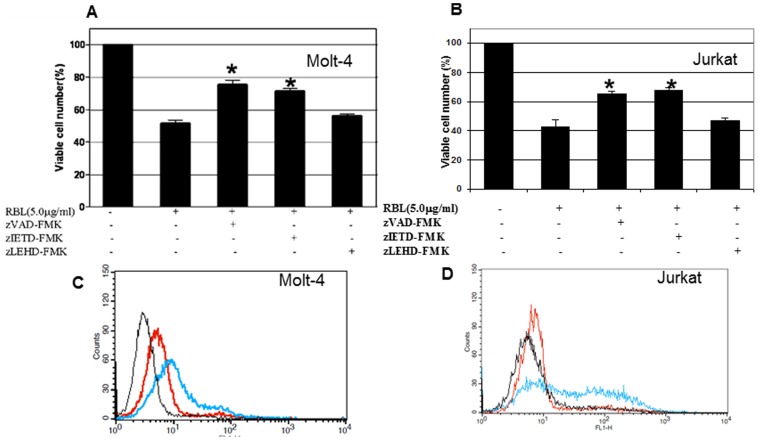
Effect of caspase-8 inhibition on RBL-induced apoptosis. Molt-4 (A) and Jurkat cells (B) were treated with RBL(5 µg/ml) for 12 h in the presence or absence of caspase inhibitors(40 µM) zVAD-FMK (pan caspase inhibitor), zIETD-FMK (caspase-8 inhibitor), or zLEHD-FMK (caspase-9 inhibitor) and viability was assessed by MTT assay. The graphs depict mean±SE values from three independent experiments. *p value <0.05 in comparison with untreated controls. Molt-4 (C) and Jurkat (D) cells were treated with RBL (5 µg/ml) for 12 h in the presence or absence of caspase-8 inhibitor (zIETD-FMK) and caspase-3 activation was assessed using flow cytometry. The overlay depicts profile of untreated cells (black line), cells pretreated with caspase-3 inhibitor followed by RBL exposure (red line) and cells treated with RBL alone (blue line).

To delineate the mechanism(s) of RBL-induced apoptosis, we analysed the effect of RBL on activation of initiator caspases-8 and -9. The cells were treated with RBL for different time periods and the activity of these caspases was measured in a colorimetric assay. The time course profiles showed a difference in the caspase-8 and caspase-9 activation. A 1.5- fold increase in caspase-8 activity was observed in cells treated with RBL for 6 h with a further increase to 1.8-fold at 12 h and was maintained till 24 h (Fig. 5A). A 1.3 fold increase in caspase-9 activity was observed 12 h post treatment with no further increase up to 24 h (Fig. 5B). To study the activation of apoptotic cascade in more detail, the activation of caspase-3 was measured by flow cytometry using a FITC tagged inhibitor that binds to active caspase-3. As shown in Fig. 5C a significant shift in the peak representing an increase in caspase-3 activity was observed in RBL-treated cells compared to control. To further determine the role of caspases in RBL-induced apoptosis, we treated Molt-4 and Jurkat cells with Z-IETD-FMK (caspase-8 inhibitor), Z-LEHD-FMK (caspase-9 inhibitor) and a pan-caspase inhibitor- Z-VAD-FMK for 1 h followed by treatment with RBL for 12 h. The MTT assay was performed to assess the cell death in RBL-treated cells occurring in the presence and the absence of inhibitors. Pretreatment with either Z-VAD-FMK, a pan-caspase inhibitor, or Z-IETD-FMK, an inhibitor of caspase-8, significantly suppressed RBL-induced cell death. The reasons for not achieving complete protection may be attributed to a small percentage of cells undergoing necrosis and secondly, the concentration of inhibitor (highest non-toxic) used may not be sufficient to block the high level of caspase activity following RBL exposure. Pretreatment with Z-LEHD-FMK, an inhibitor of caspase-9, did not significantly affect RBL-induced cell death (Fig. 6 A and B). To confirm the role of caspase-8 in RBL-induced apoptosis, caspase-3 activation was measured in the presence and absence of a caspase-8 inhibitor in Molt-4 and Jurkat cells (Fig. 6 C and D). The flow cytometry data revealed that caspase-3 activity was decreased significantly upon caspase-8 inhibition. These results pointed to the involvement of caspase-8 in RBL induced apoptosis.

### Truncation of BID and Loss of Mitochondrial Membrane Potential (MMP)

**Figure 7:**
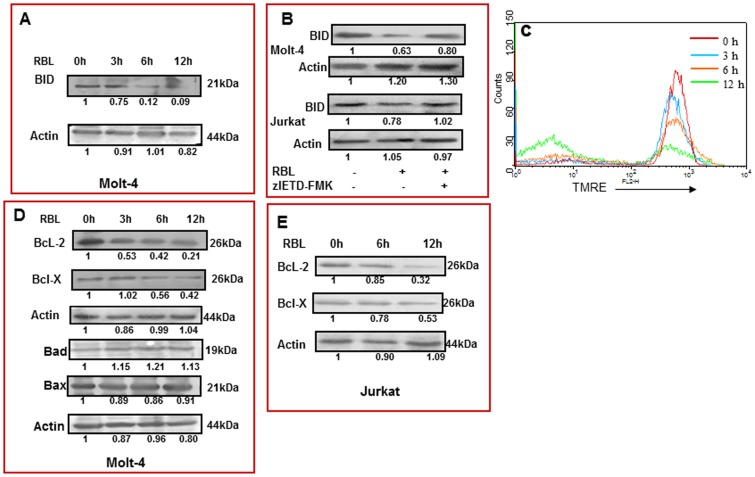
Cleavage of BID, loss of MMP, change in expression of anti- and pro-apoptotic proteins. (A) Molt-4 cells were treated with RBL (5 µg/ml) for different time periods and the effect on 21kDa uncleaved Bid was studied by western blot analysis. Actin was used as loading control. The fold differences were compared to 0 h control. (B) Molt-4 and Jurkat cells were treated with RBL(5 µg/ml) for 12 h in the presence or absence of caspase-8 inhibitor (zIETD-FMK) and truncation of BID was assessed using western blot analysis. (C) Molt-4 cells treated with RBL for different time periods –0 h (red line), 3 h (blue line), 6 h (orange line), 12 h (green line), were incubated with TMRE dye for 20 min prior to harvesting and flow cytometry analysis was performed. Loss in mitochondrial membrane potential was recorded as decrease in the red fluorescence (MFI) on FL-2 channel. The overlay is representative of three similar experiments. (D) Molt-4 cells were treated with RBL (5 µg/ml) for different time durations and the expression of Bcl-2, Bcl-X, Bad and Bax were determined by western blot analysis. (E) Jurkat cells were exposed to RBL (5 µg/ml) for different time durations and the expression of Bcl-2 and Bcl-X was assessed. Actin was used as loading control. The fold differences were compared to 0 h control.

In the extrinsic pathway, stimulation of the death receptor leads to activation of the initiator caspase-8, which then triggers downstream events either by directly activating caspase-3 or by cleavage of Bid, which in turn initiates the mitochondrial pathway [20]. A significant decrease in the expression of native 21 kDa Bid was seen in Molt-4 cells treated with RBL (5 µg/ml) for 6 h and 12 h (Fig. 7A) suggesting its cleavage, thus implicating a role of Bid in RBL-induced apoptosis. Inhibition of caspase-8 prevented truncation of BID suggesting the involvement of caspase-8 in RBL-induced truncation of Bid (Fig. 7B).

The effect of RBL on the mitochondrial membrane potential was studied using TMRE –a dye that aggregates specifically in healthy mitochondria and fluoresces in the red range. The decrease in the red fluorescence is indicative of loss of MMP. A significant decline in MMP was observed from 6 h post-treatment with further loss occurring at 12 h (Fig. 7C), suggesting that RBL treatment results in significant loss of mitochondrial membrane potential in a time dependent manner.

### Bcl-2 Family Proteins in RBL-induced Apoptosis

The mitochondrial pathway of apoptosis is regulated by members of the Bcl-2 family of proteins [21]. The involvement of Bcl-2 family members in RBL induced apoptosis was determined in Molt-4 cells. Following RBL treatment (5 µg/ml), Bcl-2 and Bcl-X expression were decreased at 3 h and 6 h, respectively, and further down regulation was observed at 12 h. Interestingly, the expression of pro-apoptotic proteins Bad and Bax were not altered till 12 h of treatment (Fig. 7 D). Furthermore, experiments in Jurkat cells also revealed the down regulation of anti-apoptotic proteins Bcl-2 and Bcl-X upon RBL treatment (Fig. 7E). These data lead us to conclude that RBL decreases the expression of anti-apoptotic proteins without affecting the pro- apoptotic proteins thereby tilting the balance towards cell death.

### Effect of RBL on the HuT-78 Cell Line

**Figure 8:**
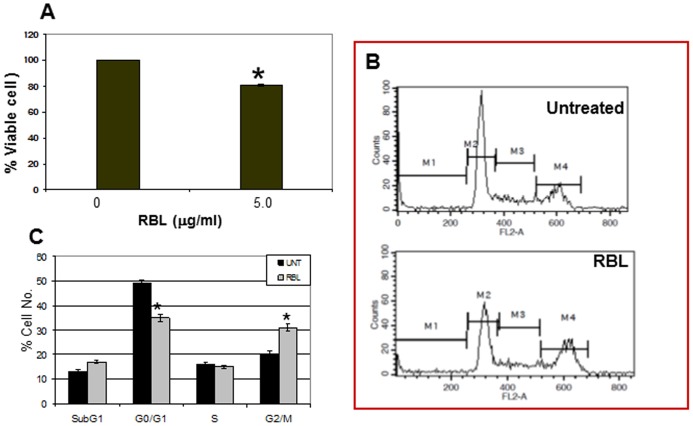
Effect of RBL on HUT-78 cells. (A) HUT-78 cells were exposed to RBL (5 µg/ml) and cell viability was assessed by MTT assay. The absorbance of untreated cells was considered as 100 to calculate percent viable cell number. The values presented in the graph are mean±SE of three independent experiments done in triplicates. *p<0.05 difference compared to untreated cells. (B) Cells treated with RBL were stained with PI and cell cycle analysis was done with data acquired on FL2-A channel. The X-axis represents the DNA content of the cells and the Y-axis represents the cell number. (C) The graph depicts the percentage of cells in different phases of cell cycle. The graph represents mean ±SE of three independent experiments. *p<0.05 significant difference between untreated and RBL-treated cells.

To address the question whether the effect of RBL was restricted to Molt-4 and Jurkat cells which are T lymphoblastic leukemia cell lines and correspond to cells arrested at relatively early stages of T cell development, we examined the effect of RBL on HuT-78 cells which is a mature human T-helper cell (CD4+ve) leukemia cell line. RBL significantly reduced the viability of HuT-78 cells (Fig. 8A) and cell cycle analysis revealed accumulation of cells in the G2/M phase (Fig. 8B and C).

### Effect of RBL on Normal T Lymphocytes and CD34+ve Hematopoietic Stem and Progenitor Cells (HSPCs)

**Figure 9:**
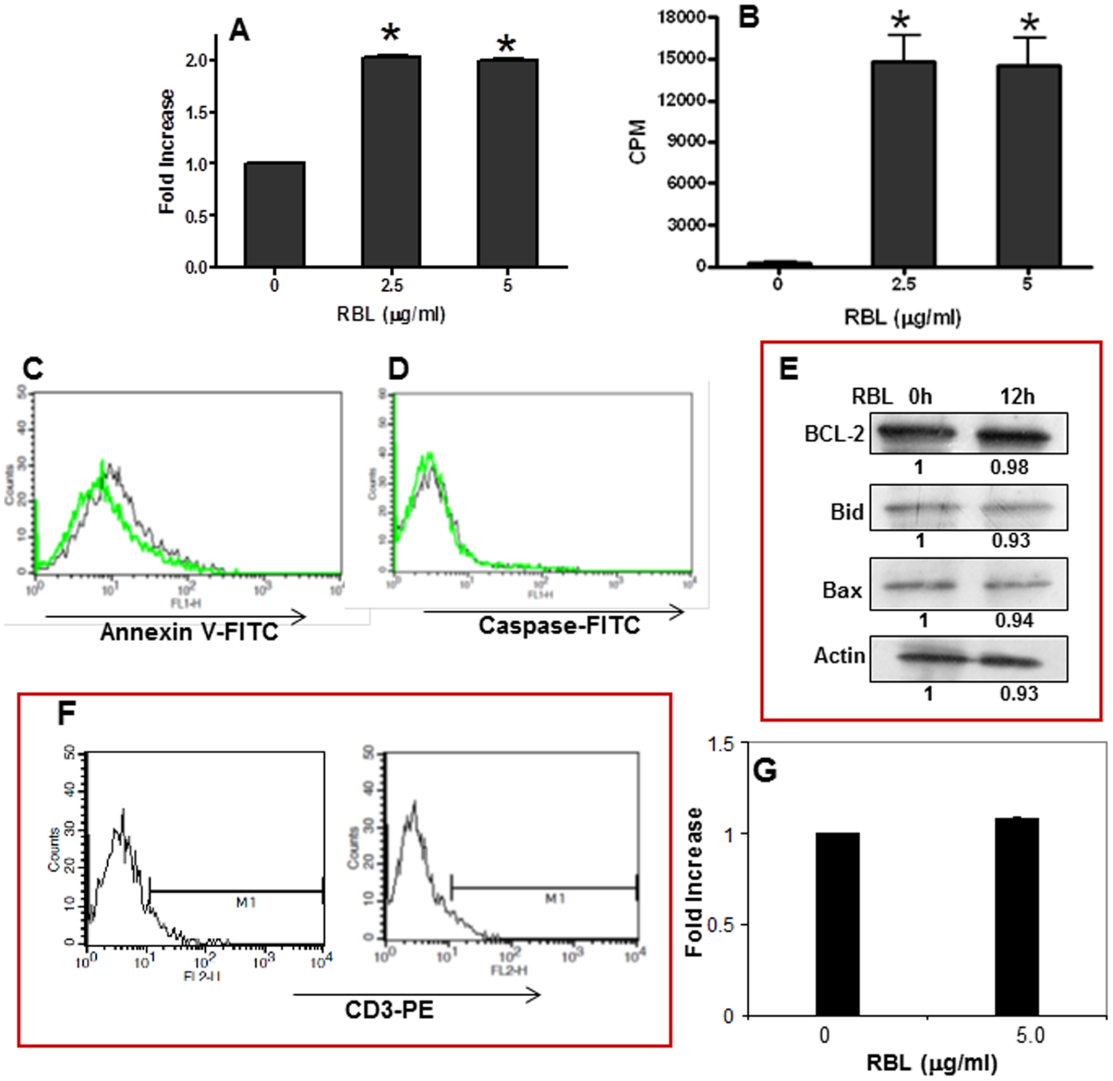
Effect of RBL on CD3 positive T-cells and CD34+ve hematopoietic stem and progenitor cells (HSPCs) cells. The response of RBL in T-cells was determined by MTT assay. (A) The graph shows RBL concentration on X-axis and fold increase on Y-axis. Fold increase was calculated by considering the absorbance of untreated cells as 1. (B) The graph depicts the concentration of RBL on X-axis and thymidine incorporation measured as counts per minute (CPM) on Y-axis. The graphs represent mean±SE from three independent experiments done in triplicates. *p<0.05 in comparison with untreated controls. PBMC were dual stained with CD3 antibody tagged with phycoerythrin and Annexin-V FITC (C) and FITC tagged caspase–3 inhibitor (D). The overlays (C and D) depicts the profiles of untreated cells (black line) and RBL-treated cells (green line). (E) PBMC were treated with RBL (5 µg/ml) for 12 h and the expression of Bcl-2, Bid and Bax was determined by western blot analysis. Actin was used as loading control. The fold differences were compared to untreated control. (F) HSPCs were stained with PE tagged anti-CD3 antibody. The histoplots depicts the absence of CD3 positive cells in the population. (G) HSPCs were exposed to RBL (5 µg/ml) for 24 h and cell viability was assessed by MTT assay. The values presented in the graph are mean+SE of three independent experiments done in triplicates.

To study the response of RBL in T-cells, CD3+ve cells were isolated by FACS sorting. A 95% pure population of T-cells was obtained in the current experimental system. The response induced by RBL in normal purified T-cells was assessed by the MTT and tritiated thymidine incorporation assay. Results from the MTT assay showed that RBL (2.5 µg/ml and 5 µg/ml) induced a 2 fold increase in the number of viable cells (Fig. 9A). To assess the proliferative potential of RBL, tritiated thymidine incorporation assay was performed by incubating the T-cells with RBL for 72 h. RBL induced a marked stimulatory effect on the proliferation of purified T-cells (Fig. 9B). To validate the finding that RBL does not induce apoptosis in untouched T-cells (T-cells not labeled with CD3 antibody), PBMC were exposed to RBL for 12 h and Annexin-V binding and caspase-3 activity were determined in gated CD3 positive population. RBL did not induce Annexin-V binding or caspase-3 activation in normal cells (Fig. 9 C and D) nor affect the expression of pro and anti-apoptotic proteins in PBMC suggesting that RBL did not induce apoptosis in normal T-cells (Fig. 9E). The effect of RBL was also assessed in normal hematopoietic stem progenitor cells (HSPCs) derived from human umbilical cord blood. The flow cytometry histoplots confirmed that the cells do not express the mature T-cell marker- CD3 (Fig. 9 F) and more importantly, RBL did not affect the viability of HSPCs suggesting that RBL is not cytotoxic to progenitor cells (Fig. 9G).

## Discussion

In this study, we investigated the anticancer activity of a fungal lectin, RBL, against human leukemia cell lines. RBL exhibited binding to the surface of leukemic cells that was inhibited in presence of complex glycoproteins but not simple sugars suggesting that RBL demonstrated specificity towards complex sugar moieties expressed on the surface of leukemic cells.

RBL significantly inhibited proliferation and induced apoptosis in the human leukemic cell lines- Molt-4 and Jurkat. Apoptosis is an organized mode of cell death that mostly involves the extrinsic/death receptor-mediated or the intrinsic/mitochondria-mediated pathway [22]. Caspase-8 is primarily activated in response to external signals that subsequently cleaves and activates the executioner caspases resulting in apoptosis [23]. Caspase-8 is frequently down regulated in leukemia that results in chemotherapy resistance and impaired prognosis in patients [24]–[26]. Hence, the majority of cytotoxic drugs routinely used in the treatment of leukemia exerts a therapeutic effect by activation of caspase-8. Caspase-8 is also activated independently of extrinsic apoptosis signaling by chemotherapeutic drugs such as chlorambucil, fludarabine and also in case of radiation-induced apoptosis [27]. Our data revealed that RBL treatment significantly induced caspases–8 activation and inhibition of caspase-8 prevented cell death. Caspase -9 was also activated but to a lesser extent compared to caspase-8 and interestingly inhibition of caspase -9 did not protect the cells from apoptosis. These findings suggest that caspase-8 plays an important role in RBL-induced apoptosis in human leukemic cells.

Caspase-8, if formed in large amount, can directly cleave effector caspases but in case of low production of caspase-8 the intrinsic mitochondrial pathway is activated through cleavage or truncation of BID [20]. RBL-induced apoptosis was associated with decreased expression of native 21kDa Bid, indicating its cleavage. Truncated BID migrates to the mitochondria and compromises the membrane integrity [28], [29]. In this context, RBL treatment resulted in a significant loss in the mitochondrial membrane potential suggesting the involvement of the mitochondrial apoptotic pathway. Depolarization of mitochondrial membrane has been documented in murine fibrosarcoma and breast cancer cells treated with the Polygonatum cyrtonema lectin and a hemagglutinin isolated from *Phaseolus vulgaris* [30], [31]. Loss of mitochondrial membrane potential results in the release of several mitochondrial intermembrane space proteins such as cytochrome c, adenylate kinase-2, and apoptosis inducing factor [32]; it is therefore essential for the cell to keep a tight control over mitochondria and this rigorous check is maintained by Bcl-2 family members. Lectins such as Abrus agglutinin [33] and Polygonatum cyrtonema lectin [34] have been reported to down regulate the expression of anti-apoptotic Bcl-2 family members in the human cervical cancer cell line HeLa and a melanoma cell line, respectively. Exposure to RBL resulted in decreased levels of the anti-apoptotic proteins Bcl-2 and Bcl-X without affecting the pro-apoptotic proteins–Bad and Bax thereby shifting the balance towards cell death. It is not the expression of these proteins *per se* but the ratio of pro- and anti-apoptotic proteins that decides the fate of a cell. An altered ratio of pro- and anti-apoptotic Bcl-2 family members is reported in various human malignancies due to overexpression of anti-apoptotic proteins [35]. Therefore, agents targeting anti-apoptotic Bcl-2 family members have clinical significance when used alone or in combination with other antineoplastic agents [36], [37]. Down regulation of anti-apoptotic Bcl-2 family of proteins can improve susceptibility to apoptosis and thus overcome resistance to cancer chemotherapy [38]. To further address whether the effect of RBL was restricted to Molt-4 and Jurkat cells which represent immature T -lymphoblastic leukemia cell lines, we examined the effect of RBL in a mature T-helper cell leukemia cell line - HuT-78. RBL induced inhibition of proliferation in HuT-78 cells with arrest of cells in the G2/M phase suggesting differential responses of leukemic cells arrested in different stages of T- cell development. Anticancer agents such as etoposide [39], diosgenin [40] and sulforaphane [41] have been reported to induce arrest in G2/M phase in leukemic cells.

To finally ascertain that the cytotoxicity induced by RBL was specific to transformed cells, it was important to determine its effect on normal T-cells. To this end, interestingly, we found that RBL did not induce apoptosis in T-cells sorted using CD3 antibody but on the contrary increased the proliferation as reported earlier for PBMC [17]. Also, RBL did not induce apoptosis in T-cell population in normal PBMC thus ruling out the involvement of CD3 antibody in the experimental system. Importantly, to confirm that RBL did not adversely affect the progenitor population, we also tested the effect of RBL on immature hematopoietic stem and progenitor cells (HSPCs) that do not express the mature T-cell antigen -CD3. RBL did not affect the viability of these cells suggesting that RBL does not induce cytotoxicity in normal T-cells or in the progenitor cells. Though lectins such as Phaseolus vulgaris lectin have been shown to induce proliferation in mouse splenocytes and cytotoxicity in tumor cell lines [42],there are no reports of differential responses of normal and leukemic T-cells in different stages of development. Based on these findings and our earlier *in vitro* and *in vivo* studies demonstrating RBL-induced cytotoxicity in ovarian cancer cells [43] we suggest that RBL can be explored as a potential candidate for cancer therapy.
